# A novel wearable device for automated real-time detection of epileptic seizures

**DOI:** 10.1186/s42490-023-00073-7

**Published:** 2023-07-17

**Authors:** Mikael Habtamu, Keneni Tolosa, Kidus Abera, Lamesgin Demissie, Samrawit Samuel, Yeabsera Temesgen, Elbetel Taye Zewde, Ahmed Ali Dawud

**Affiliations:** School of Biomedical Engineering, Jimma Institute of Technology, Jimma University, Jimma, Ethiopia

**Keywords:** Acceleration, Epileptic seizure, Jerky movement, Oxygen denaturation, Real-time detection, Wearable sensors

## Abstract

**Background:**

Epilepsy is a neurological disorder that has a variety of origins. It is caused by hyperexcitability and an imbalance between excitation and inhibition, which results in seizures. The World Health Organization (WHO) and its partners have classified epilepsy as a major public health concern. Over 50 million individuals globally are affected by epilepsy which shows that the patient’s family, social, educational, and vocational activities are severely limited if seizures are not controlled. Patients who suffer from epileptic seizures have emotional, behavioral, and neurological issues. Alerting systems using a wearable sensor are commonly used to detect epileptic seizures. However, most of the devices have no multimodal systems that increase sensitivity and lower the false discovery rate for screening and intervention of epileptic seizures. Therefore, the objective of this project was, to design and develop an efficient, economical, and automatically detecting epileptic seizure device in real-time.

**Methods:**

Our design incorporates different sensors to assess the patient’s condition such as an accelerometer, pulsoxymeter and vibration sensor which process body movement, heart rate variability, oxygen denaturation, and jerky movement respectively. The algorithm for real-time detection of epileptic seizures is based on the following: acceleration increases to a higher value of 23.4 m/s^2^ or decreases to a lower value of 10 m/s^2^ as energy is absorbed by the body, the heart rate increases by 10 bpm from the normal heart rate, oxygen denaturation is below 90% and vibration should be out of the range of 3 Hz -17 Hz. Then, a pulsoxymeter device was used as a gold standard to compare the heart rate variability and oxygen saturation sensor readings. The accuracy of the accelerometer and vibration sensor was also tested by a fast-moving and vibrating normal person’s hand.

**Results:**

The prototype was built and subjected to different tests and iterations. The proposed device was tested for accuracy, cost-effectiveness and ease of use. An acceptable accuracy was achieved for the accelerometer, pulsoxymeter, and vibration sensor measurements, and the prototype was built only with a component cost of less than 40 USD excluding design, manufacturing, and other costs. The design is tested to see if it fits the design criteria; the results of the tests reveal that a large portion of the scientific procedures utilized in this study to identify epileptic seizures is effective.

**Conclusion:**

This project is objectively targeted to design a medical device with multimodal systems that enable us to accurately detect epileptic seizures by detecting symptoms commonly associated with an episode of epileptic seizure and notifying a caregiver for immediate assistance. The proposed device has a great impact on reducing epileptic seizer mortality, especially in low-resource settings where both expertise and treatment are scarce.

## Background

Epilepsy is a neurological disorder that has a variety of origins. It is caused by hyperexcitability and an imbalance between excitation and inhibition, which results in seizures [1, 2]. Epileptic seizures are jerky or trembling body movements caused by abnormal neural activity, which can injure the brain or other organs [3]. Even a single seizure can disrupt brain development and cause behavioral and cognitive problems. Seizures have a substantial impact on patient’s lives, leading to emotional, behavioral, and neurological issues. Seizures can occur in several locations of the brain, and treatment effectiveness varies based on the individual area, seizure type, and location of abnormal neuronal activity [4].

The World Health Organization (WHO) and its partners have classified epilepsy as a major public health concern worldwide that has a major impact on developing countries. According to the WHO, epilepsy affects over fifty million people globally, making it one of the most common neurological illnesses. The seemingly unexpected unpredictability of seizures is one of the most frustrating elements of epilepsy [5–9]. If seizures are not controlled, the patient’s family, social, educational, and vocational activities are severely limited. Furthermore, a life-threatening condition in which seizure activity occurs continuously is frequently treated successfully only with drastic intervention. Until now, the medical profession held the idea that epileptic seizures could not be predicted. Seizures were thought to be sudden transitions that occurred at random. However, hypotheses based on reports from clinical experience and scientific intuitions, such as “Lennox’s ‘reservoir theory”, have suggested that seizure prediction may be possible.

Prediction of epileptic seizures in early detection might help in the prevention of sudden unexpected deaths in epilepsy (SUDEP), which is a condition that might cause a person to enter a life-threatening condition during seizure episodes. According to current studies, the majority of occurrences of SUDEP are during or shortly after a seizure. Although the specific cause is unknown, apnea, problems related to the heart that can lead to cardiac arrest, and other mixed conditions are among plausible causes [10].

There are two types of excitatory and inhibitory impulse transmission. The former is facilitated by the neurotransmitter gamma-aminobutyric acid (GABA), which functions as the primary inhibitory agent in the brain through the mediation of chloride and potassium channels [11]. After being triggered, the impulses travel through the neuronal circuits along the nerves’ axons. The action potential then proceeds down the axon to the terminal buttons, which subsequently discharge neurotransmitters into the synaptic cleft [12]. In normal circumstances, genetic mutations, trauma, abnormal development, or a variety of other stressors disrupt inhibitory interneurons that regulate the excitatory synaptic activity, resulting in hyperexcitable cortical networks [13]. Even if some special engineering equipment dynamic characteristics and vibration laws are not researched as clearly as other mechanics, the existing research shows the important parts of the human body vibration frequency are generally located in approximately 3–17 Hz [14–17].

The primary cause of the high incidence of epilepsy in developing nations is primarily attributed to inadequate obstetric care, which results in an increased likelihood of perinatal brain injury. Other contributing factors include the effects of cerebral malaria, complications of endemic parasites, infectious diseases, fever, head trauma, injuries related to poverty, and other chronic ailments affecting the brain [18].

The seemingly unexpected unpredictability of seizures is one of the most frustrating elements of epilepsy. Even a single seizure can disrupt brain development and cause behavioral and cognitive problems. If it is not controlled, the patient’s family, social, educational, and vocational activities are severely limited.

## Method

### The proposed design

The proposed device is designed based on four basic scientific research characteristics of epileptic seizures: occurrence of fall which is detected by the accelerometer; oxygen denaturation is below 90% for most seizure cases; heart rate variability, in which ictal tachycardia is very common phenomenon during seizure episodes. It is the increase in heart rate by 10 bpm from the normal heart rate of the patient and Jerky movement or vibration is a condition that occurs during tonic-clonic seizures.

To assess the patient’s condition the system incorporates different sensors. The system obtains information about the seizure episode by analyzing four inputs. Heart rate and oxygen saturation data are taken using a MAX30100 sensor, after which a heart rate variability algorithm checks for the existence of ictal tachycardia which is a very common condition during epileptic seizures and is characterized by an increase in the normal heart rate baseline of the patient over 10 bpm. The second input is body orientation which is detected using accelerometer data in which a patient’s hand acceleration is measured for the detection of falls during seizures. This is done by taking the average acceleration of the hand and comparing it to a threshold value in which the value exceeds that threshold and is characterized as a fall. The last input is jerky movement in which a simple vibration sensor is used to detect its occurrence by measuring fluctuating accelerations or speeds or for normal vibration measurement. A vibration sensor is a type of accelerometer but an accelerometer is not necessarily a vibration sensor. An accelerometer measures the quality of acceleration, not necessarily vibration or jerky movements. In vibration, three basic characteristics of jerky movements are measured to evaluate the vibration that describes the movement: namely frequency, amplitude and acceleration.

During some types of seizures especially tonic-clonic seizures, there is an abrupt shaking movement of the body and limbs. Thus, the vibration sensor can detect this shaking and check the threshold value and monitor if the measured value exceeds the presented value.

After gathering different sensory data a decision algorithm on the microcontroller will evaluate the data and give detection results. The mobile app was developed using the MIT app inventor2 online app maker, in which an Android app can be made using dedicated blocks and drag and drop design. The mobile app has a very simple interface in which it receives a signal from the Arduino Uno and sends the location and state of the patient through email and gives a signal by changing color which is green for false alarm and red for danger alert. These final data will then be sent to the patient’s smartphone using wireless Bluetooth Low Energy (BLE) technology. Finally, the smartphone will send an alert message to the specified person responsible for the patient. In addition, the system also contains two buttons one is used for sending a false alarm message when the system wrongly characterizes the patient condition, and the other is used as a panic button where the user can send a message if he/she needs help.

The final prototyped design goes through several processes and iterations. The initial step was to gather and purchase the materials needed for the design and to install software and programming tools for simulating the design. The second stage is developing the algorithm and writing code for Proteus, which is a software embodiment of the design and then applying it to Arduino programming software. The third stage was to construct the basic electrical part of the design on a simple breadboard including sensors and other components. After designing the casing part on the AutoCAD hardware design, an improved prototype is built in a defined structure using cardboard, and tests were done on the device. Then the final design is well painted and designed utilizing 3D printed materials that offer the design additional qualities such as hardness, durability, and attractiveness.

The final design of the device was the way the system sends an alert message to the responsible body which is achieved by using a Bluetooth module to send the processed sensor data to the patient’s smartphone and in turn, the phone will send an automated email to the caregiver based on the data it receives from the Arduino microcontroller. This is done using the patient’s smartphone, which already contains a location sensor, which is a more convenient solution than employing a GPS module interfaced with an Arduino Nano making the system bulkier and less portable.

**Fig. 1:**
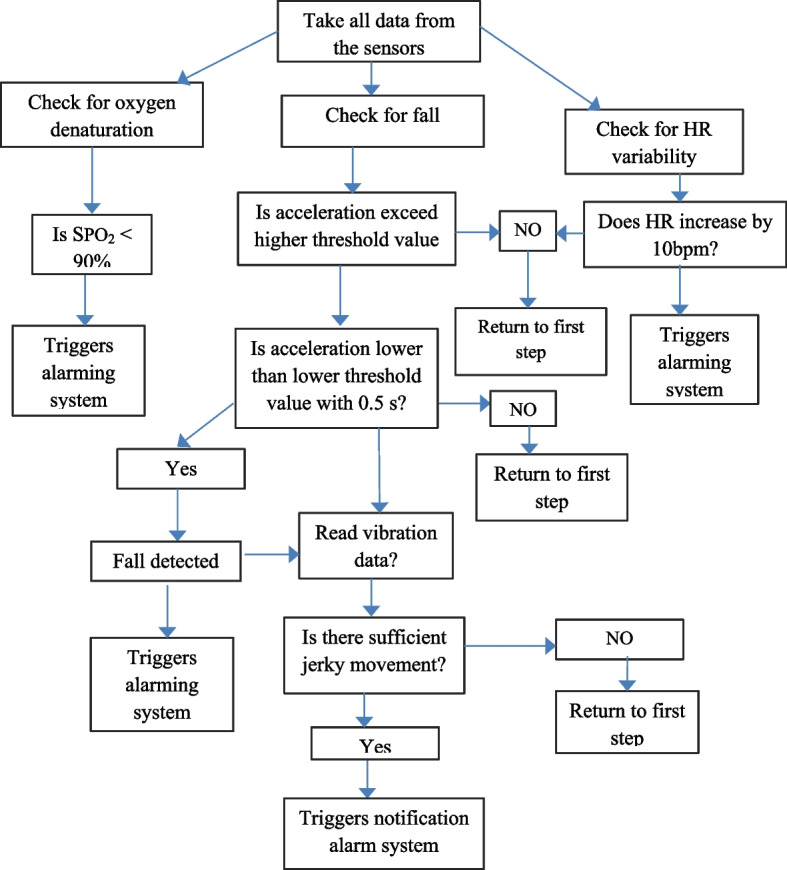
Functional and general block diagram of the proposed algorithm

The algorithm for real-time detection of epileptic seizures is based on the fact that acceleration increases to a higher value and immediately returns to a lower value because the energy is absorbed by the body. A higher threshold value of 23.4 m/s^2^ and a lower threshold value of 10 m/s^2^ for position (body orientation) and a higher threshold value of 17 Hz and a lower threshold value of 3 Hz for vibration are used. The threshold value for heart rate is 10 bpm which is a very common phenomenon during seizures and is known as ictal tachycardia and SpO2 is shown to decrease below 90% during seizure sessions, as shown in Fig. 1.

### Materials used

Table 1 demonstrates the materials and their specifications used to construct the prototype.

**Table 1:** List of materials and specifications used to construct the prototype

S. No	Items	Specification
1	Arduino	Arduino Nano
2	OLED display	SSD1306, 0.96inch
3	Jumper wire	-
4	Micro push button	12 V
5	LED	5 V
6	Bluetooth module	HC-05
7	Heart rate sensor	MAX30100
8	Vibration sensor	SW-420
9	Buzzer	5 V
11	Accelerometer	ADXL337
12	Resistor	1kohm,300Ω,4.7kohm
13	3D printed casing	PVC

## Results

### Simulation result

**Fig. 2:**
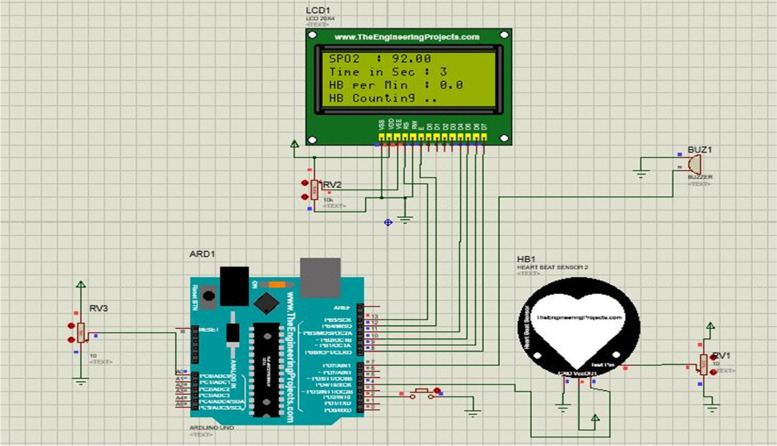
Heart rate simulation

**Fig. 3:**
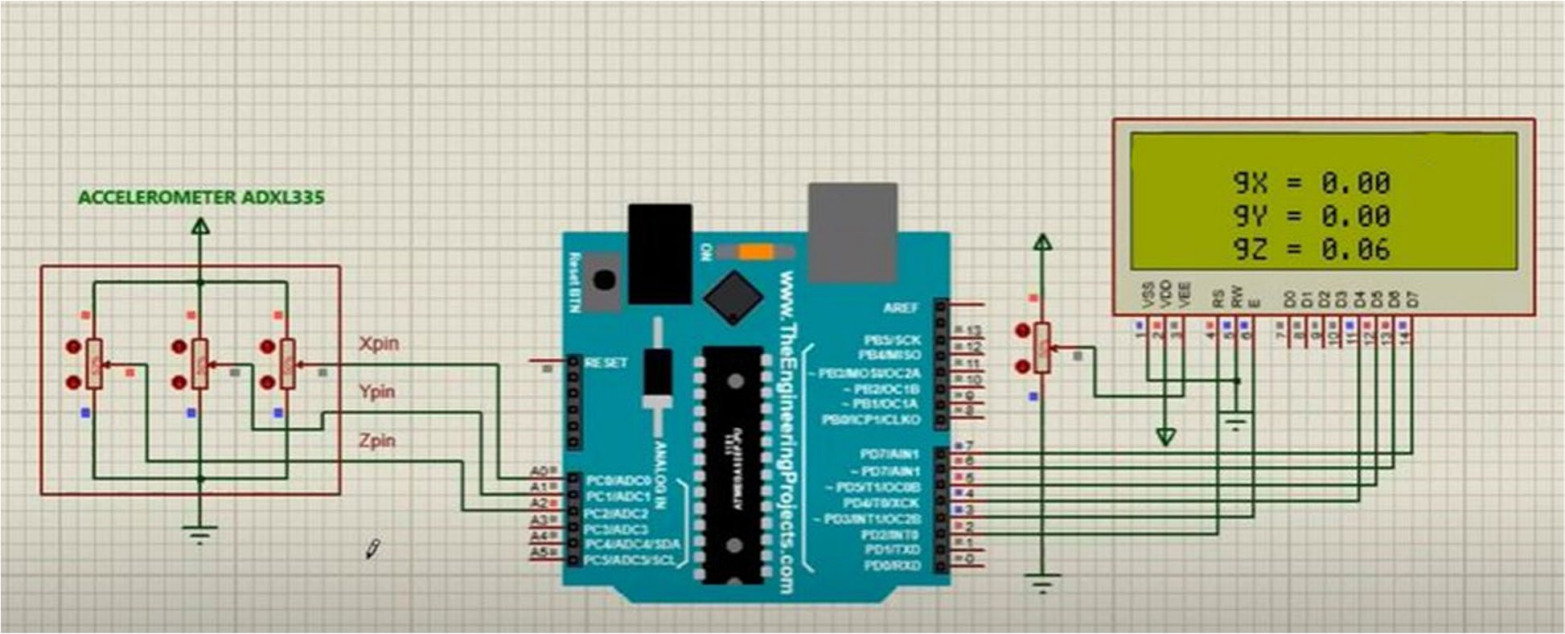
Accelerometer simulation

**Fig. 4:**
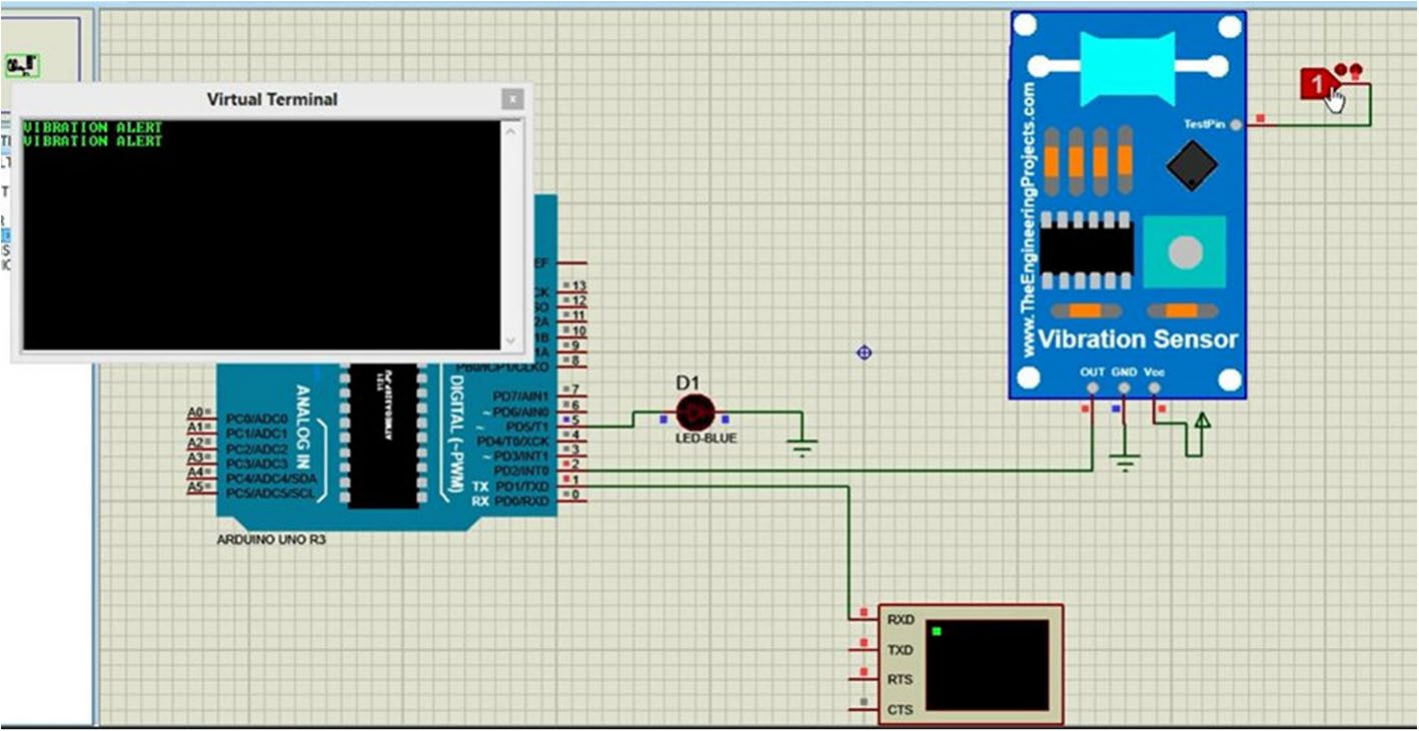
Vibration sensor simulation

The design was simulated using Proetus software and Arduino Uno prior to prototype construction and real testing. The inputs used for the simulated system were the heart rate sensor which is to the digital pin of the Arduino Nano but since there was no sensor library for measuring oxygen concentration a potentiometer connected to the Arduino analog pin was used for simulating purposes. The final output was displayed using an LCD and a buzzer was used for simulating the alarming part, as shown in Fig. 2. To simulate the measurement of accelerometer data, potentiometers were used because of a lack of a library. The three potentiometers connected to the Arduino analog pins simulate three-dimensional accelerometer data and were displayed on the LCD, which can be used to calculate the patient’s body acceleration, as shown in Fig. 3. The last simulation was to simulate measuring vibration and alarm in which a vibration sensor was connected to digital pin 2, and the data were displayed using an LCD, as shown in Fig. 4.

### Prototype iterations

**Fig. 5:**
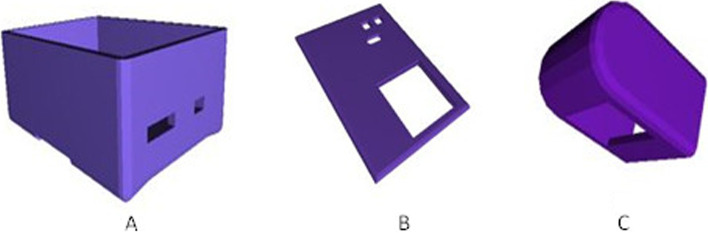
3D design of the proposed device: **A** Box, **B** Lid, **C** finger house

**Fig. 6:**
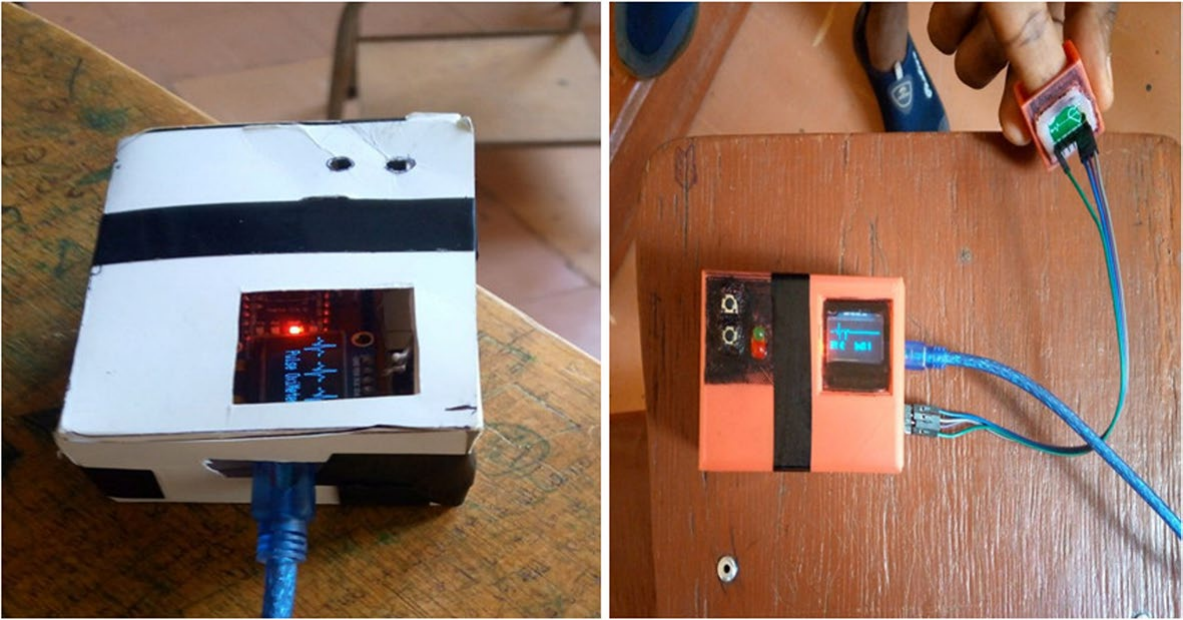
The first and final prototype

Several iterations of prototypes have been undertaken to enhance our design. The 3D design of the proposed device and the finger house is illustrated in Fig. 5, while Fig. 6 displays the initial and final constructed prototypes. The initial prototype was constructed using waste materials like cartons. However, after testing, the entire body of the device was constructed using 3D printing.

### Prototype test results

The design criteria used to design and construct the prototype were accuracy, safety, cost, portability, and ease of use. The test results were conducted against the design criteria. Accordingly, the accuracy of the prototype units was checked by performing different tests. The accuracy of heart rate and oxygen saturation sensors was tested by using a normal person. Then, a pulsoxymeter device was used as a gold standard to compare with the sensor’s readings. Following the same procedure, the accuracy of the accelerometer and vibration sensor was also tested by fast moving and vibrating a normal person’s hand. Testing was conducted for ten iterations, and an average accelerometer and vibration sensor accuracy of 90.2% was acquired. Table 2 demonstrates the testing methods and test results obtained.

**Table 2:** Test methods and test results

S.No	Design criteria to be tested	Method	Result
1	Heart rate, Accuracy	By using a normal person and observing the reading signal	Accuracy: 98%
2	accelerometer and vibration sensor	By fast movement and vibration of a normal person’s hand	Accuracy: 90.2%
3	Cost	Component cost	Approximately 4000 Ethiopian Birr (ETB)
4	Portability	Weight measuring	200 g
5	Easy to use	Operating procedure	15 min training for the user and caregiver

**Fig. 7:**
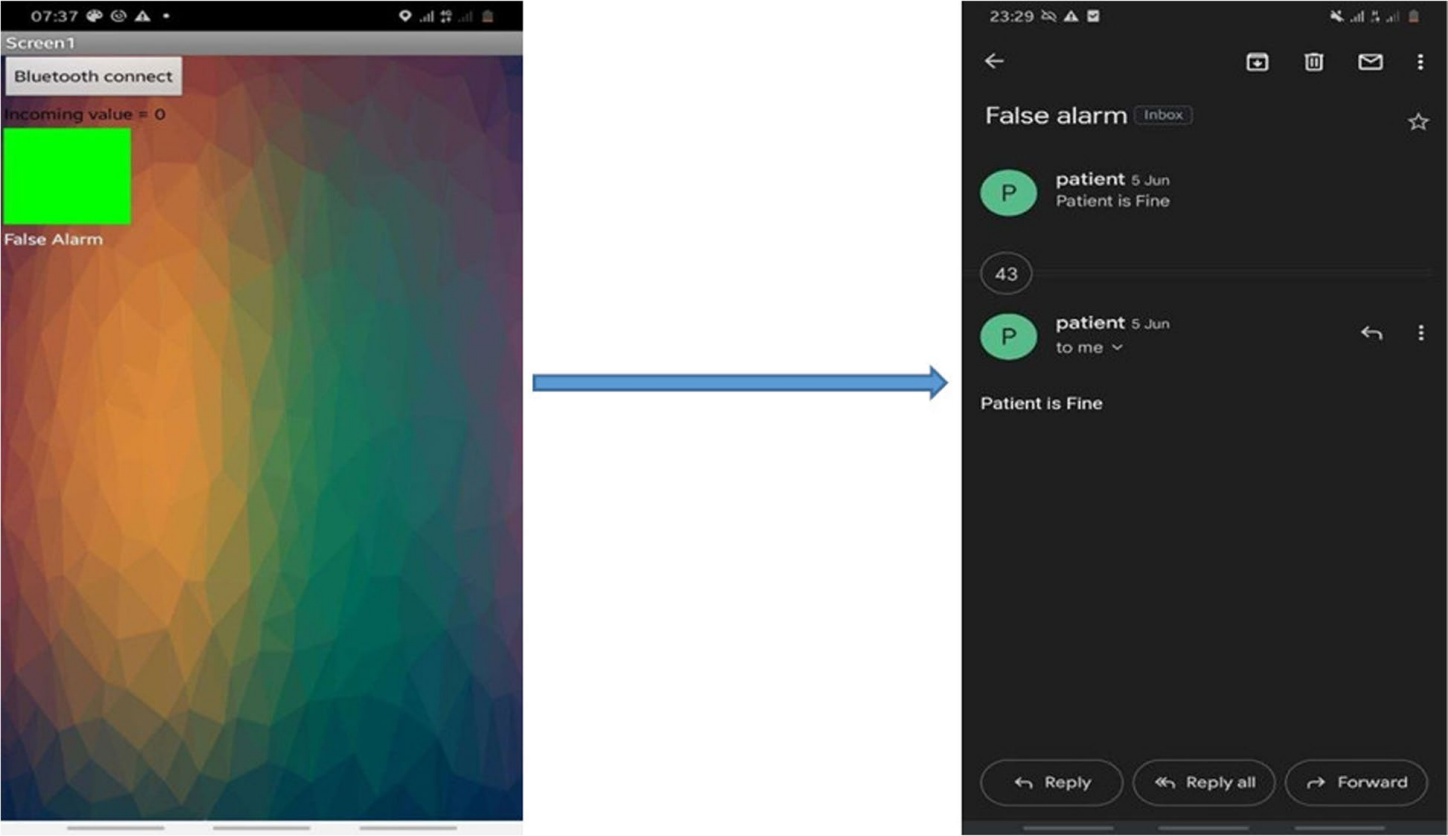
Mobile application interface

The mobile app has a very simple interface in which it receives a signal from the Arduino microcontroller, sends the location and state of the patient through email, and gives a signal by changing color. The green is for the false alarm and the red is for the danger alert as shown in Fig. 7.

## Discussion

Epilepsy seizures have complicated pathophysiology, which explains the great spectrum of seizure diseases. All biochemical, structural, and functional changes are present at the beginning and evolution of the patient’s epilepsy. It remains the most prevalent, neglected, and serious neurological disorder as well as one of the major causes of disability in most counties worldwide. This implies that the problem requires much attention. To solve the detection problem some existing solutions, such as EEG, surface electromyography, implanted advisory systems, magnetometers, and accelerometers have been designed. However, these devices and tests are limited to solving the problem as expected.

The hardware section is made up of several components, including an accelerometer for detecting falls during epileptic seizure episodes and a heart rate and oxygen saturation sensor for detecting heart rate variability and oxygen desaturation that occurs during seizures. This is done by placing the hand in the 3D printed compartment, which will help to understand and analyze the state of the patient. The other is a vibration sensor, which will help in the detection of jerky movement after a fall has occurred. The system also contains two buttons: one is used for sending a false alarm message when the system wrongly characterizes the patient condition, and the other is used as a panic button where the user can send a message if he/she needs help. The other part is an OLED display screen that displays the patient heart rate and oxygen saturation in real time.

The device can perform a variety of tasks including detecting symptoms caused by an episode of epileptic seizures. It can detect whether the person is at risk of an epileptic seizure or not and can work anywhere without selecting environments. The microcontroller has built-in ADC converters. Direct microcontroller interfaces have been used for good resolution and high noise levels. A microcontroller with sensors determines the charging or discharging time of an RC circuit. This time-to-digital conversion is affected by the quantization of the timer and the trigger noise, which limit the resolution to an effective number of bits.

The device that is used to detect falling, jerky movement, heart rate, and oxygen concentration of the patient can be used: In developing countries such as Ethiopia and other African countries where epilepsy is very high, it can give prediction results for the prevention and management of patients. Any individual epileptic patient can afford the device and use it to know their seizure condition since it is simple and easy to use everywhere. The designed device also has a great impact in minimizing the workload for the healthcare giver or other supporters who need to give support to the patient, reducing the number of epileptic patients who are going to suffer from falling in Ethiopia.

Our proposed solution is unique because of the following: firstly, it is designed as a small watch-like device that is comfortable for the patient to wear; secondly, it includes false alarm detection to prevent unnecessary alerts during non-seizure incidents such as falls or obstacles; thirdly, the device incorporates heart rate and oxygen saturation sensors to monitor the patient’s vital signs without interfering with the fall detection mechanism. And lastly, the device has a built-in battery for convenience. The proposed method is first simulated before building the prototype. The sensor readings and the controller’s feedback or regulation mechanism were evaluated using a simulator and the system was modified accordingly. The body of the final design is built using 3D printing. The design has been designed to be simple and user-friendly so that patients and caregivers can easily adapt to the proposed system without much training. The components used to build the prototype cost less than US$40 (excluding design, manufacturing, and other costs), making it potentially affordable for countries with low resources. The accuracy of the sensors used has been gold standard tested and the average accuracy is 90.2% and 98% for oxygen saturation and heart rate sensors, accelerometer and vibration sensor respectively.

As there was no real-world testing of the device, tests on the animal model should be done before clinical testing and evaluation on humans. In this work, we have demonstrated a potentially effective low-cost cooling device that could, with more evaluation, be effective as a means of detecting epileptic seizures. In addition, the proposed design introduces the importance of twitching or jerking muscle movements, uncontrolled tightening of muscles, or repetitive/automatic movements for the detection of epileptic seizures measured by vibration sensors.

## Conclusion

In this work, a device was developed by considering significant parameters including body movement, heart rate variability, oxygen denaturation and jerky movement, and many studies have shown that these parameters have significant value to detect epileptic seizures. Therefore, based on these scientific facts a device was designed to measure the patient’s heart rate, body acceleration, oxygen denaturation, and jerky or shaking movement. Most parameters are acquired using different types of sensors, such as accelerometers for body acceleration, pulsoxymeter for heart rate, and oxygen denaturation and vibration sensors for jerky movement detection. The average accuracy of the heart rate variability and oxygen denaturation sensor readings was 98%, while that of the accelerometer and vibration sensor readings was 90.2%. Our preliminary test results show that the proposed low-cost device is promising and, with more evaluation, can be used as an effective detection and monitoring method for epileptic patients. This will have a great impact on reducing epileptic seizer mortality, especially in low-resource settings where both expertise and treatment are scarce.

Finally, the fall and jerky movement detection system will be implemented using a sensor containing both an accelerometer and gyroscope, such as MPU6050, by reducing the complexity and size of the algorithm to be implemented using a simple single-board microcontroller or a simpler solution using an integrated sensor for fall and jerky movement detection.

## Data Availability

The datasets used and/or analyzed during the current study are available from the corresponding author on reasonable request.
